# The Relationship Between Social Communication and Social Functioning in Pediatric TBI: A Pilot Study

**DOI:** 10.3389/fneur.2019.00850

**Published:** 2019-08-14

**Authors:** Helen M. Genova, Alison Haight, Joman Y. Natsheh, John DeLuca, Jean Lengenfelder

**Affiliations:** ^1^Kessler Foundation, East Hanover, NJ, United States; ^2^Department of Physical Medicine and Rehabilitation, Rutgers-New Jersey Medical School, Newark, NJ, United States; ^3^Children's Specialized Hospital Research Center, New Brunswick, NJ, United States

**Keywords:** pediatric, traumatic brain injury, social communication, social cognition, theory of mind

## Abstract

**Objective:** Social communication presents a significant difficulty for children with traumatic brain injury (TBI). Although several measures are used to examine social communication, there is no gold-standard assessment tool. The present pilot study examined the ability of the Social Communication Disorders Checklist (SCDC) to detect social communication difficulties in pediatric TBI. Further, we examined the relationship between social communication and social functioning as assessed by parental ratings of behavior and objective measures of social cognition.

**Methods:** Sixteen children with pediatric TBI and 20 age, education and sex matched healthy controls (HCs) participated. All participants participated in a neuropsychological evaluation and parents filled out questionnaires. Parents rated their children's social communication abilities using the SCDC, as well as the Behavior Assessment System for Children, Second Edition (BASC-2). The pediatric subjects completed a task of social cognition, specifically Theory of Mind (ToM).

**Results:** The pediatric TBI group had significantly lower scores on the SCDC compared to the HCs (*p* = 0.001). In the pediatric group, SCDC scores correlated significantly with scores on the BASC-2, as well as performance on the ToM task, indicating that children with lower parent-rated social communication abilities also had lower scores on the objective measure of social cognition.

**Conclusions:** These data provide preliminary evidence that children with TBI have difficulties with social communication, as evidenced by lower scores on the SCDC, and that SCDC scores correlate with subjective and objective measures of social cognition and behavior in pediatric TBI.

## Introduction

Traumatic brain injury (TBI) that occurs during childhood results in cognitive, behavioral, emotional and social impairments (1–3). Such impairments can persist for years, even into adulthood (3–5). Pediatric survivors of TBI have significant impairments in “social functioning” (1, 2, 6), a term which refers to “the way an individual operates in a social environment by relying on social skills and interacting with others” (7). Impaired social functioning is associated with reduced well-being, academic performance and community integration (8, 9). Given its impact on other aspects of life, thorough investigation and assessment of social dysfunction in pediatric TBI is needed in clinical practice.

Social functioning can refer to a range of skills, including social or emotional perception, social cognition, social skills, social behavior, among others (7). One impairment particularly affected in persons with TBI is social communication. A recent meta-analysis indicated that a number of social communication skills are affected by pediatric TBI, including: taking-turns, maintaining a topic, discussion of appropriate topics, discourse organization, comprehension of contextual language (understanding ironic, deceptive or sarcastic language), perspective-taking, and understanding of non-verbal cues (10). Multiple theories of social functioning have suggested that social communication is also highly related to skills involving social cognition, including Theory of Mind (ToM) (7, 11, 12). ToM is the ability to understand the thoughts and beliefs of others, even when those thoughts/beliefs may not be obvious (such as the use of sarcasm). In the current study, we examined social communication in pediatric TBI utilizing the Social Communication Disorder Checklist [SCDC; (13)], and whether social communication assessed by the SCDC is related to social cognition, namely ToM.

Although social communication deficits have been studied extensively in pediatric TBI (10, 14–16), studies on social communication in pediatric TBI have relied on a number of varying measures, suggesting that there is no “gold-standard” for measuring this deficit (10). Many screening tools are cumbersome for parents to fill-out, and/or validated on a restricted age range [e.g., items on the La Trobe Social Problem Skills Inventory (17) may not be appropriate for younger children]. Thus, it has been suggested that more research is needed to accurately assess social communication in pediatric TBI, specifically examining possible screening tools (10). The SCDC is a unique measure that comprises the domains of (1) social reciprocity, (2) non-verbal skills (3) pragmatic language usage, and (4) behavioral impairments that affect interactions. It was developed as a quick and sensitive screening tool for autistic traits in children (13), as well as a measure of social communication abilities in clinical populations of attention-deficit-hyperactivity disorder (18) and oppositional defiant disorder (19). To date, however, its utility has not been examined in pediatric TBI.

In the current study, we sought to utilize the SCDC to examine social communication issues in pediatric TBI. The objective of the current study was to examine social communication using the SCDC to compare children with TBI to healthy controls (HC's). We examined whether difficulties quantified on the SCDC would be associated with parental ratings of other types of social functioning skills, such as those measured on the Behavior Assessment System for Children, Second Edition (BASC-2). Because the SCDC is a parent-report measure (and therefore subjective), we further investigated whether the SCDC was correlated with objective (non-self/parent report) measures of ToM: Ironic Criticism and Empathetic Praise task (ICEPT) a task which has been shown to be impaired in children with TBI. (11, 20). It was hypothesized that children with TBI who have more difficulty in social communication also have worse social cognitive abilities and social functioning.

## Materials and Methods

### Participants

Participants included 16 children with a diagnosis of moderate-to-severe TBI. Injury severity was confirmed through parent-report in addition to a review of medical records. Specifically, diagnostic criteria included GCS scores (if available) of 8–12 (moderate), or <8 (severe), loss of consciousness (LOC) of >30 min (moderate) or >24 h (severe), as well as additional evidence of neurologic injury based on imaging (see Table 1).

**Table 1:** Demographics in children with TBI and HCs and injury variables.

**Demographics**	**TBI (*n* = 16)**	**HCs (*n* = 20)**
Age (Mean ± SD)	11.56 ± 3.69	11.05 ± 3.11
Education (Mean ± SD)	6.19 ± 3.62	5.45 ± 3.07 years
Sex	11/16 Male	9/20 Male
Injury type		n/a
Motor Vehicle Accident (MVA)	12 (75%)	
Fall	1 (6.3%)	
Sports	2 (12.5)	
Abuse	1 (6.3%)	
Injury severity		n/a
Moderate	8 (50%)	
Severe	8 (50%)	
Months since injury		n/a
Mean (SD)	64.88 (44.28)	
GCS		n/a
Mean (SD)	6.43 (4.28)	
Positive for LOC	14 (87.5%)	n/a
Positive for imaging findings	12 (75%)	n/a

*Education was defined in years (first grade completed = 1 year, second grade completed = 2 years, etc.); GCS, Glasgow Coma Scale; LOC, Loss of Consciousness*.

Recruitment was achieved through Children's Specialized Hospital and through local neurology clinics. TBI participants were included if they were between the ages of 7 and 18, were at least 1 year post injury, and spoke English fluently. Participants were excluded based on a history of any neurological condition aside from the brain injury, a psychological disorder or learning disability diagnosed prior to the injury, or substance abuse. An additional 20 pediatric participants were included as HCs, and were matched with TBI participants for age. The control group was recruited through local schools, or were relatives of those in the TBI group. There were no significant group differences found in education [*t*_(34)_ = 0.66, *p* = 0.232, *d* = 0.22 or age: *t*_(34)_ = 0.45, *p* = 0.188, *d* = 0.15] or sex between TBI and HC groups, *X*^2^ = 2.031, *p* = 0.154. A summary of the demographic and injury specific variables is included in Table 1.

If participants in this study were 18 years old, they signed an informed consent approved by the Institutional Review Board of Kessler Foundation. If participants were minors, parents completed a consent for their child. All children were present during the consent process, and signed either a consent (children ages 13–17) or assent form (children ages 7–12). Compensation of $50 was provided to participants involved in this research.

### Measures

Eligibility was first determined based on a parental phone screen, which detailed demographics, injury history, and related medical information. The measures used in this study were part of a larger battery completed by participants and their parents.

### Social Functioning

#### Social Communication Disorders Checklist (SCDC)

Social Communication Disorders Checklist (SCDC) (13) was completed by parents/caregivers, and consisted of 12 items describing potential social communication skills of their child over the past six months. Parents rated how strongly the item applied to their child. The maximum score was 24, suggesting high social difficulties, while the lowest score was zero, or no noticed difficulties. The questions of the SCDC measure (1) social reciprocity (5 questions); (2) non-verbal skills (1 question); (3) pragmatic language usage (3 questions); and behavioral impairments that affect interactions (3 questions).

#### BASC-2

BASC-2 (21) is a standardized questionnaire used to assess skills, adaptive behaviors, and problematic behaviors or personality traits in children. The current study utilized the Parent Rating Scales (PRS)—Child (ages 6–11; PRS-C) and Parent Rating Scales—Adolescent (ages 12–21; PRS-A). Parents answered 160 questions for the PRS-C, or 150 questions for the PRS-A, indicating how frequently each behavior occurs in their child at home or in the community by rating it as “never,” “sometimes,” “often,” or “always.” For this study, we converted raw scores to T scores, and then created composite scores for each category: Externalizing Problems, Internalizing Problems, Behavioral Symptoms Index, and Adaptive Skills. Lower scores on all composite scores indicated less impairment, aside from Adaptive Skills in which a higher score indicates higher functioning in this area.

#### The Ironic Criticism and Empathetic Praise Task (ICEPT)

The Ironic Criticism and Empathetic Praise Task (ICEPT) (22) measures Theory of Mind, specifically, children's ability to recognize and interpret intentionality and inflection (22). ICEPT included 18 trials in which a scenario is described with a corresponding picture. In each scenario there was an image of an individual completing a task either poorly or well. There was also a second individual (the speaker) in each scenario who comments on how well the task was done. Participants were told the speaker's intentions (e.g., “He liked to cheer people up” or “He liked to bug and annoy people”). Participants then listened to a recording of the speaker telling the actor how well the task was done (e.g., “You did a great job tidying your room”). The inflection of the speaker's voice indicated whether the speaker was speaking literally (honestly), empathetically, or sarcastically. Participants were asked questions to assess story comprehension, as well as the ability to detect the beliefs (e.g., “*What did Jim think about the cake”)*, and intentions *(e.g., “What did Jim want Betty to think about the cake”)*, behind the speaker's comment. The dependent variables for ICEPT were the following for the current study: a total score (total items correct across all trials), a mastery score (children receive one point toward their Mastery Score for each story in which correct answers are given for all four belief and intention questions), total score for all belief questions, and total score for all intention questions. The IECPT has been shown to be impaired across multiple studies of pediatric TBI (11, 20, 22).

### Statistical Analyses

Data analysis was conducted using Statistical Package for the Social Sciences for Windows, Version 21 (SPSS). Independent sample *t*-tests were used to compare TBI participants with HC participants on age, sex and education. To examine group differences between the TBI and HC groups on the SCDC, ICEPT, and the BASC-2, multivariate analyses of variance were run. Because the age range was broad in the current sample, and because social communication and social cognition is affected by development, we include age as a covariate. One child in the pediatric TBI group did not complete the BASC-2 due to time constraints during testing session. Pearson correlations were run to examine the relationship between SCDC and all other social functioning measures in the TBI group only, as our research question was specific to the TBI group. As age of injury is likely a confounding variable, we also performed partial correlations, controlling for age of injury.

## Results

### Group Differences on SCDC, BASC-2, and ICEPT

Group differences are summarized in Table 2. On the SCDC, parents of children with TBI rated their children as having significantly higher social communication problems compared to HCs (*p* < 0.001). According to parental ratings on the BASC-2, the pediatric TBI group had significantly higher Externalizing Problems Composite scores (assessing hyperactivity, aggression, and conduct problems) compared to the HC group (*p* = 0.009). The pediatric TBI group also had significantly higher scores on the Internalizing Problems Composite Score (assessing anxiety, depression, and somatization) compared to HCs (*p* = 0.011). The pediatric TBI group had significantly higher scores on the Behavioral Symptoms Index (assessing atypicality, withdrawal, and attention problems) compared to HCs, *p* = 0.001. The pediatric TBI group had significantly lower scores on the Adaptive Skills Composite (assessing adaptability, social skills, leadership, activities of daily living, and functional communication) compared to HCs, *p* < 0.001.

**Table 2:** Group differences on tasks of social functioning.

	**TBI *n* = 16 (15 for BASC)**	**HC *n* = 20**	***F***	***p*-value**	**Partial eta squared**
SCDC	9.69 (6.44)	0.65 (1.14)	48.72	0.000^*^	0.604
BASC externalizing	55.67 (16.24)	42.70 (5.14)	12.15	0.001^*^	0.275
BASC internalizing	58.13 (18.98)	43.60 (6.20)	10.84	0.002^*^	0.253
BASC behavioral	60.33 (17.06)	41.50 (4.83)	24.24	0.000^*^	0.431
BASC adaptive skills	39.80 (9.07)	58.60 (7.10)	29.25	0.000^*^	0.478
ICEPT correct	142.13 (32.35)	171.55 (18.08)	15.50	0.000^*^	0.326
ICEPT mastery	9.31 (5.70)	14.70 (3.77)	17.11	0.000^*^	0.348
ICEPT belief	45.31 (19.10)	61.30 (9.92)	11.18	0.002^*^	0.259
ICEPT intent	44.94 (17.23)	56.40 (11.47)	7.96	0.008^*^	0.199

*SCDC, Social Communication Disorders Checklist; BASC-2, Behavior Assessment System for Children, Second Edition; ICEPT, Ironic Criticism and Empathetic Praise task*.

* *Significance levels of < 0.01*.

In terms of social cognition, the pediatric TBI group showed significant impairments relative to HCs on ICEPT, in terms of total score, *p* = 0.004, mastery scores, *p* = 0.003, all belief questions, *p* = 0.006, and all intentions questions, *p* = 0.023.

### Association Between SCDC and Subjective/Objective Measures of Social Functioning

Pearson correlations revealed that parental rating on the SCDC was correlated positively with all composite scores of the BASC-2 PRS for the pediatric TBI group: Externalizing Problems Composite [*r*_(15)_ = 0.66, *p* = 0.004, Internalizing Problems Composite [*r*_(15)_ = 0.53, *p* = 0.022], Behavioral Symptoms Index [*r*_(15)_ = 0.77, *p* < 0.001], and negatively with Adaptive Skills Composite [*r*_(15)_ = −0.76, *p* < 0.001]. These correlations indicate that parents who rated their children as having worse social communication abilities rated their children as having more behavioral and social concerns on BASC-2. Partial correlations, controlling of age since injury were consistent with bivariate correlations: SCDC scores correlated with Externalizing Problems Composite (r_partial_ = 0.63, *p* = 0.008), Internalizing Problems Composite (*r*_partial_ = 0.50, *p* = 0.035), Behavioral Symptoms Index (*r*_partial_ = 0.75, *p* < 0.001), and negatively with Adaptive Skills Composite (*r*_partial_ = −0.64, *p* < 0.007).

Related to the ICEPT task, Pearson correlations revealed that the SCDC total score was negatively correlated with the following ICEPT variables: total correct on all ICEPT questions, [*r*_(16)_ = −0.54, *p* = 0.016], ICEPT Mastery Score, [*r*_(16)_ = −0.55, *p* = 0.014], total intention questions, [*r*_(16)_ = −0.61, *p* = 0.006]. Bivariate correlations were largely consistent with the partial correlations, which revealed SCDC correlated with total correct on the same ICEPT questions, even after controlling for age of injury [*r*_partial_ (16) = −0.49, *p* = 0.038], ICEPT Mastery Score, [*r*_partial_ (16) = −0.48, *p* = 0.041], total intention questions, [*r*_partial_ (16) = −0.58, *p* = 0.015].

## Discussion

This pilot study examined social communication deficits in pediatric TBI using the SCDC, and investigated whether parent-reported difficulties in SCDC correlate with other measures of social functioning. The results showed that children with TBI have significantly lower social communication compared to HCs, offering preliminary evidence that the SCDC is able to detect these deficits in pediatric TBI. Further, the SCDC scores in children with TBI were correlated with both subjective and objective measures of social functioning and social cognition. The SCDC is a tool to assess children with autism spectrum disorder (ASD) (13, 23, 24), as well as autistic traits in the general population (25). The results of this study suggest that the SCDC may have clinical utility in children with TBI.

A number of questionnaires have been used to assess social communication impairments following pediatric TBI including the La Trobe communication questionnaire (17), the social problem solving skills inventory—revised (26) and the social skills rating scale (27). However, while these measures have been shown to be able to detect social communication impairments in TBI, we propose that the SCDC may hold some benefit over them. While some social communication measures can have as many as 57 items and take as much as 45–60 min to fill out, the SCDC is a short assessment tool with only 12 items. Thus, it can be used as a screening tool for clinicians as it is not cumbersome for parents and can be completed quickly. Utilization of such a tool would better guide clinicians to develop treatment plans incorporating ways to improve social communication (i.e., social skills therapy or metacognitive therapy) (28–30). Due to the preliminary nature of the current study and the small sample size, the findings should be interpreted with caution. More research is needed on the utility of the SCDC in pediatric TBI, including examination of psychometric properties, in order for it to be recommended as a screening tool.

The current study examined whether parental ratings of social communication assessed with SCDC were related to either subjective or objective measures of social function and social cognition. On the BASC-2, children with TBI were rated by their parents as having more behavioral and social issues compared to HCs. Further, BASC-2 ratings were significantly correlated with the SCDC ratings. As the BASC-2 assesses social functioning (including some elements of social communication), the correlation between these two measures suggests a level of convergent validity that should be confirmed with a larger sample in a psychometric study designed to test the validity of the SCDC in pediatric TBI.

As both the BASC-2 and the SCDC are based on parental report and therefore subjective in nature, the current study included a task of ToM to investigate how SCDC scores were related to objective measures of social cognition. Children with poor ToM skills, evidenced by reduced performance on ICEPT, also had reduced social communication skills. Impairments in ToM may make it difficult for some to understand the beliefs/thoughts of others, which may further lead to inappropriate social communication/behavior. In line with this, the recently postulated socio-cognitive integration of abilities model (SOCIAL) suggests that social cognitive abilities (emotion perception) can affect social skills/function, taking into account mediating factors such as brain development/health, socioeconomic status and culture (7). This model also suggests that social cognitive impairments specifically may be associated with reduced social functioning, as opposed to general cognitive abilities. The results of the current study are in line with this model in pediatric TBI.

A further possibility explaining the relationship between social communication and social cognitive abilities is that the relationship between the two deficits are due to a direct consequence of brain injury affecting neural networks responsible for both social communication deficits and ToM. Consistent with this, studies have shown that reduced integrity of the corpus callosum following pediatric TBI is associated with both ToM deficits and social communication difficulties (31). Thus, as brain regions responsible for multiple aspects of social functioning are damaged following TBI, deficits in both social communication and social cognition may occur (32). Future studies combining structural and functional neuroimaging with measures of social cognition and social communication will further elucidate the neural networks underlying these separate areas of social functioning.

A third possibility to explain the relationship between social cognition and social communication is related to the development of social functioning skills in pediatric TBI. Longitudinal studies have shown that social functioning deficits persist for years following a TBI (3, 33, 34). An injury early on in life (e.g., infancy) may have lasting effects on the social brain, especially on social perception skills which develop in infancy (ToM). However, the later development of social communication skills may be affected because either brain regions which were injured may not develop properly or the needed social cognition skills are not present at the time of communication development. Further, other factors related to social skills development may influence social communication. For example, social isolation and reduced friendship has been observed in children with TBI (35). It can be argued that a child who is socially isolated would have reduced ability to practice/develop social communication skills. At this point, future studies examining the longitudinal development of social skills over time in pediatric TBI are needed to examine whether factors such as social isolation affect social communication development.

The current study was limited by a small sample size. A larger sample size would allow for the comparison between TBI severity groups and to adequately examine the psychometric properties of the SCDC in pediatric TBI. More rigorous analyses of psychometric properties would potentially allow for recommendation of the SCDC to be utilized as a clinical screening tool. Additionally, a larger sample would enable us to perform analyses which would indicate whether the SCDC ratings predicted social communication impairments identified in a more comprehensive evaluation with standardized measures. Further, the lack of neuroimaging data does not enable examination of neural networks underlying social communication deficits in pediatric TBI. Thus, future studies combining imaging with SCDC will allow for better understanding of how certain TBI-related injuries, such as diffuse axonal injury, are related to social communication skills.

In conclusion, the present results demonstrate that children with TBI are impaired in social communication as indicated by the SCDC. As the SCDC was designed to examine deficits in ASD, these findings indicate a behavioral overlap between pediatric TBI and ASD that should be further explored. Furthermore, social communication was associated with an objective task of social cognition (ToM), supporting theoretical models that social communication is highly related to social cognition. While the current sample size precludes us from making any definitive conclusions, this study may indicate that the SCDC is a tool which can be utilized in TBI to examine social communication. Future studies should be conducted which are more highly powered to examine the psychometric properties of this task in pediatric TBI.

## Data Availability

The datasets generated for this study are available upon request to the corresponding author.

## Ethics Statement

This study was carried out in accordance with the recommendations of the institution review board (IRB) of Kessler Foundation with written informed consent from all subjects. All subjects gave written informed consent in accordance with the Declaration of Helsinki. The protocol was approved by the Kessler Foundation IRB.

## Author Contributions

HG conceptualized the study design. HG and AH wrote the first draft of the manuscript and performed statistical analysis. AH organized the database and tested subjects. HG, AH, JN, JD, and JL wrote sections of the manuscript. All authors provided editorial comments and approved the submitted version.

### Conflict of Interest Statement

The authors declare that the research was conducted in the absence of any commercial or financial relationships that could be construed as a potential conflict of interest.
